# The trajectory of conditional, recurrence-free, and long-term survival in a complete 10-year cohort of patients with advanced ovarian cancer

**DOI:** 10.2340/1651-226X.2025.42994

**Published:** 2025-03-17

**Authors:** Nina Groes Kofoed, Henrik Falconer, Matteo Bottai, Sahar Salehi

**Affiliations:** a Department of Women’s and Children’s Health, division of Obstetrics and Gynaecology, Karolinska Institutet, Stockholm, Sweden; b Department of Pelvic Cancer, Theme Cancer, Karolinska University Hospital, Stockholm, Sweden; c Division of Biostatistics, Institute of Environmental Medicine, Karolinska Institutet, Stockholm, Sweden

**Keywords:** Carcinoma, ovarian epithelial, recurrence, platinum-free interval, secondary cytoreductive surgery

## Abstract

**Background:**

The prognosis in advanced ovarian cancer is generally poor since the majority experience recurrence. Nevertheless, there is a chance of cure and very long-term survival may be achieved. However, traditional survival metrics do not account for the dynamic changes in prognosis over time. Our objectives were to examine conditional, very long-term and recurrence-free survival, in addition risk-factors for recurrence.

**Methods:**

In this observational study, all patients diagnosed with advanced ovarian cancer between 2009 and 2018 in the Stockholm/Gotland region, Sweden, were identified in the Swedish Quality Registry of Gynecologic Cancer. Conditional and recurrence-free survival were estimated with the Kaplan Meier method. The association between predefined clinical factors and hazard of death was analysed with Cox regression yielding hazard ratio (HR) with 95% confidence interval (CI).

**Results:**

A total of 888 patients were analysed of which 87.0% (*n* = 740) experienced a recurrence and 8.5 % (*n* = 76) were alive > 10 years. The 5-year conditional survival increased from 33.0% (95% CI: 30–36) in patients who had survived 1 year to 57.0% (95% CI: 50–63) in patients who had already survived 5 years. The median recurrence-free survival was 18 months (95% CI: 16–19). Risk factors associated with recurrence included any residual tumour (> 10 mm; HR: 2.15; 95% CI: 1.65 to 2.80) and evidence of disease at end of first line treatment (HR: 2.40; 95% CI: 1.97 to 2.93; *p* < 0.001).

**Interpretation:**

Conditional survival improves significantly with time survived following an advanced ovarian cancer diagnosis. Most patients experience relapse within 1 year after completing first-line treatment, nevertheless long-term survival is possible.

## Background

Ovarian cancer is the most lethal of all gynaecologic malignancies as the majority of patients are diagnosed at an advanced stage [1]. Treatment comprises a combination of surgical cytoreduction, chemotherapy, and targeted therapies when indicated [1, 2].

While prognosis is generally poor, very long-term survival (>10 years) may be achieved [2–8]. Survival estimates calculated from the time of diagnosis hold limited relevance for patients who have already survived several years, as these analyses account only for individuals who remain alive at a specific time point after diagnosis. Conditional survival, meaning that the time already survived is taken into account when estimating the probability of further survival, may provide more accurate information on current prognosis for the individual patient and caregiver alike.

Despite a seemingly successful primary treatment, in complete remission, most patients with advanced ovarian cancer recur [9–12]. In the recurrent situation, treatment is not considered curative and comprises the same alternatives as in the first line. However, the choice of including platinum based cytotoxic agents in second line treatment depend on the time passed between the relapse and last dose of adjuvant chemotherapy during first line treatment (platinum free interval) [1, 9].

Oncologic outcomes in advanced ovarian cancer may vary substantially across health care systems and settings, as patient selection to treatment differs [13–18]. Nevertheless, data on conditional, recurrence-free and very long-term survival are limited and altogether lacking from a public and centralised health care system setting. Accordingly, detailing these data, would contribute to an improved patient counselling and an individualised surveillance strategy as the benefit of current follow-up (including physical exams, imaging and monitoring of CA-125 levels) is limited [19].

For this reason, our objective was to investigate the conditional and very long-term survival in advanced ovarian cancer. Moreover, to examine the recurrence-free survival and risk factors for recurrence.

## Patients and methods

This was a registry-based observational study which included all patients with International Federation of Obstetrics and Gynecology (FIGO) stages III and IV epithelial ovarian cancer, diagnosed between 2009 and 2018 in a public and heavily centralised health care system in the Stockholm/Gotland Region of Sweden.

The Stockholm Ovarian Cancer Project (STOOVCA) database and studies from the database were deemed exempt from review by the Regional Ethics Committee at Karolinska Institutet (Dnr: 2016-1233-21/4).

### Database and patients

Data were retrieved from the STOOVCA database, where inclusion- and exclusion criteria, registries, validation, and treatment have been described in detail previously [13]. In brief, patients with FIGO stages III and IV epithelial ovarian/fallopian tube/peritoneal cancer (defined as ICD-O-2 topographical codes; C76.2 [cancer abdominis], C76.3 [cancer pelvis], C48.1 [primary peritoneal cancer], C48.2 [unspecified peritoneal cancer], C57.0 [fallopian tube cancer], and C56.9 [ovarian cancer]; and ICD-O-2 morphologic codes; 8020/3, 8140/3, 8310/3, 8380/3, 8440/3, 8441/3, 8450/3, 8460/3, 8461/3, 8470/3, 8980/3, and 9014/3) in the Region of Stockholm/Gotland in Sweden were identified through the Swedish Quality Registry for Gynecologic Cancers (SQRGC) with 100% coverage against the Swedish National Cancer Registry. All patients received treatment at Karolinska University Hospital, the only tertiary referral centre for gynaecologic oncology in the region. The STOOVCA database entails completely validated (against source data; hospital records) and monitored data on patient-, treatment-, and tumour characteristics, including recurrence and vital status.

### Inclusion criteria

International Federation of Obstetrics and Gynecology stages III and IV epithelial ovarian cancer patients who underwent surgery with the intention of cytoreduction, diagnosed between 2009 and 2018, and received treatment at Region Stockholm/Gotland, Sweden were included in the study.

### Exclusion criteria

All procedures which fell under the category of non-surgical treatment and/or non-elective surgery (emergency surgery) were excluded from the study.

### Outcome measures

Conditional overall survival was defined as the probability of further survival depending on years already survived.

Very long-term survival was defined as survival >10 years after diagnosis.

Recurrence-free survival (includes progression free survival and death) and Recurrence (includes progression) were defined by the following hierarchy: (1) histologically proven relapse (date of biopsy), (2) relapse upon imaging (date of computer tomography), (3) elevated CA-125 (date of examination), and (4) symptomatic progression (date of clinical assessment).

Vital status and recurrence were estimated from date of diagnosis to date of recurrence, death or for patients still alive without recurrence to 2024-06-20.

### Covariates to investigate risk-factors for recurrence

Risk-factors included age, FIGO stage, American Society of Anaesthesiologists physical status classification (ASA-score), timing of surgery, extent of cytoreductive surgery (surgical complexity score), residual tumour, number of cycles of chemotherapy, complete remission at end of first line treatment [20].

### Statistical analysis

Descriptive statistics are reported with medians and interquartile range (IQR) or with numbers and proportions, as appropriate.

Conditional overall and recurrence-free survival were estimated with the Kaplan Meier method. Survival time after recurrence was calculated from the date of recurrence to the date of death, and for patients still alive, until the date of data collection on vital status. Survival functions were tested for equality with the log-rank test.

The association between predefined clinical factors and recurrence-free survival was analysed with Cox regression. The model included age (< 60, 60–69, ≥ 70), FIGO stage (III vs. IV) ASA-score (I–II vs. III–IV), timing of surgery (upfront vs. interval), surgical complexity score (low 0–3, medium 4–7, high ≥ 8), residual tumour (0 mm, ≤10 mm, > 10 mm), number of cycles of chemotherapy (6 , < 6, > 6), complete remission after first line treatment (yes vs. no). The proportional hazards assumption was tested in all included variables, and if the assumption was not met, the variable was included as strata in the final model. The results from the model were presented as hazard ratio (HR) with associated 95% confidence interval (CI), and Wald’s test *p*-values.

Follow-up time was estimated with the Kaplan Meir method. Completeness of data was estimated with Clark’s completeness index [21].

The significance level was set at 5%, and all *p*-values reported are two-sided.

Statistical analyses were performed with the statistical software Statistical Package for the Social Sciences (SPSS) version 29 and Stata (Stata Corp, College Station, United States) version 16.

## Results

**Figure 1:**
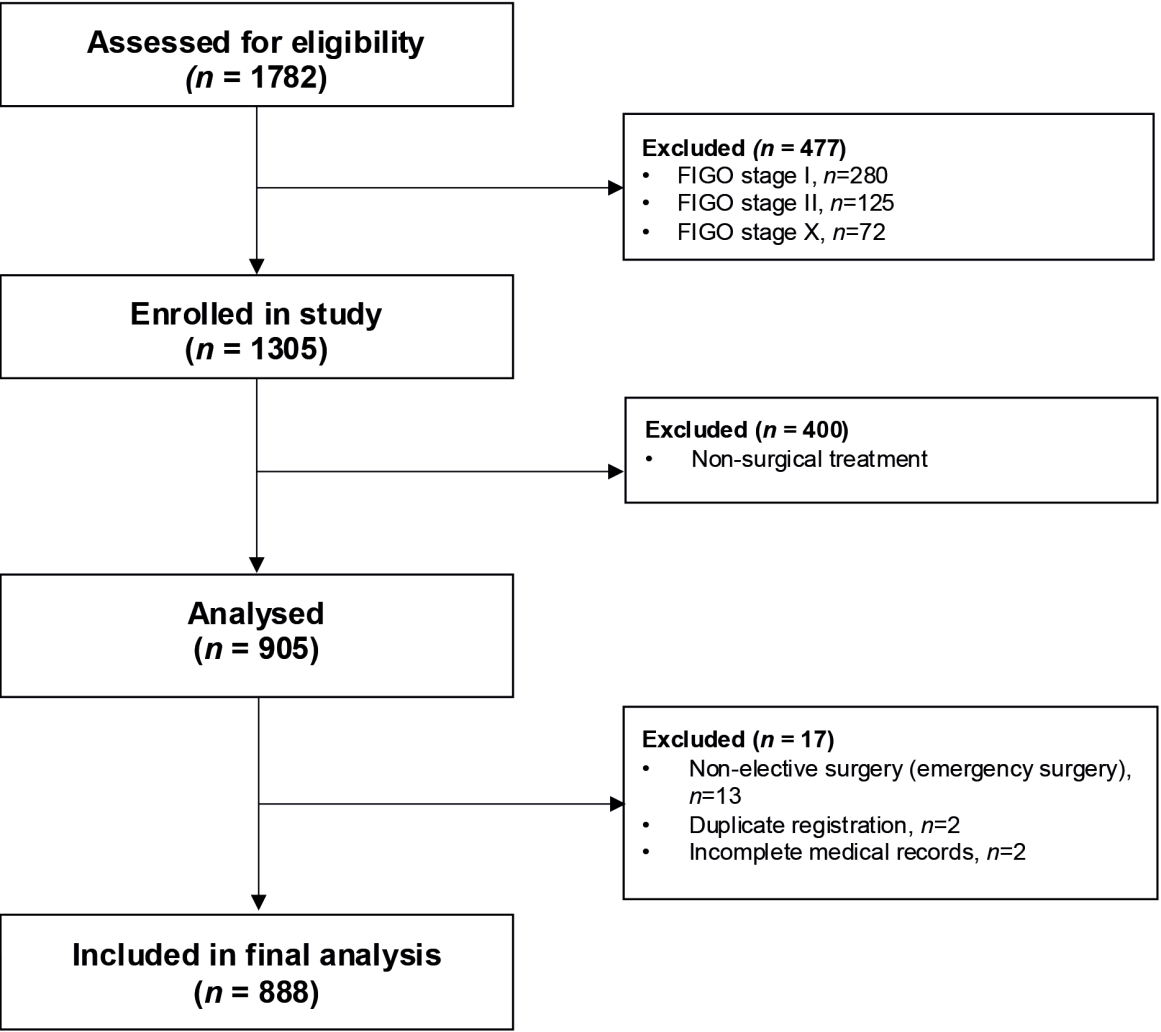
Flowchart over the selection of patients for analysis from the Swedish Quality Registry of Gynecologic Cancer. FIGO: International Federation of Gynecology and Obstetrics; CRS: cytoreductive surgery; FIGO stage X refers to patients in whom stage was not reported to the SQRGC.

Between 2009 and 2018, a total of 1,782 patients were identified in the SQRGC and assessed for eligibility. After exclusion of patients with FIGO stages I, II, and X (*n* = 477), non-surgical treatment (*n* = 400), non-elective surgery (*n* = 13), duplicate registration (*n* = 1), and not treated in the Stockholm/Gotland Region, Sweden (*n* = 2), 888 patients were included in the analysis (see Figure 1).

Baseline characteristics are presented in Table 1. The median age at diagnosis was 66 years (IQR: 56–72), the majority were diagnosed in FIGO stage III (69%, *n* = 611), received upfront surgery (75%, *n* = 664), and had complete macroscopic resection (58%, *n* = 513). Only 9% (*n* = 75) of the patients received fewer than six cycles of chemotherapy. At end of first line treatment 59% (*n* = 523) were assessed as in complete remission (see Table 1).

**Table 1:** Clinical and treatment characteristics of patient diagnosed with advanced ovarian cancer between 2009 and 2018 in the Stockholm/Gotland Region, Sweden.

Characteristics	*n* = 888
Age, years	
Median (IQR^*^)	66 (56–72)
Age, years group, no. (%)	
≤ 59	289 (33)
60–69	298 (34)
≥ 70	301 (34)
FIGO stage, no. (%)	
III	611 (69)
IV	277 (31)
ASA-score, no. (%)	
I–II	542 (61)
III–IV	323 (36)
Missing	23 (3)
Timing of surgery, no. (%)	
Upfront	664 (75)
Interval	224 (25)
Surgical complexity score^a^, no. (%)	
Low (0–3)	387 (44)
Intermediate (4–7)	231 (26)
High (≥ 8)	268 (30)
Missing	2 (0)
Residual tumour, mm, no. (%)	
0	513 (58)
1–10	163 (18)
> 10	208 (23)
Missing	4 (1)
Number of cycles of chemotherapy, no. (%)	
< 6	75 (9)
6	507 (57)
> 6	305 (34)
Missing	1 (0)
Complete remission at end of first line, no. (%)	
Yes	523 (59)
No	361 (41)
Missing	4 (1)

IQR: Interquartile range; FIGO: International Federation of Gynecology and Obstetrics; ASA-score: American Society of Anaesthesiologists physical status classification.

* Refers to 1st to 3rd quartile

a According to Aletti et al. [20].

The median observed follow-up time (time from inclusion to end of study, death or loss to follow-up) was 43 months (min. to max. 1–182) (see Supplementary Table 1).

The conditional survival by each year already survived is presented in Table 2. The 1-year conditional survival rate was similar the first 5 years, between 77% and 82%. The 3-year conditional survival rate increased from 49% (95% CI: 46 to 52) after 1 year survived to 67% (95% CI: 61 to 72) after 5 years survived. The 5-year conditional survival rate increased from 33% (95% CI: 30–36) in patients who had survived 1 year to 57% (95% CI: 50–63) in patients who had already survived 5 years.

**Table 2:** Conditional survival of patient diagnosed with advanced ovarian cancer between 2009 and 2018 in the Stockholm/Gotland Region, Sweden.

		Time already survived (*n*)				
		1 year (*n* = 817)	2 years (*n* = 670)	3 years (*n* = 516)	4 years (*n* = 402)	5 years (*n* = 327)
Conditional survival rate % (95% CI)	1-year	82 (79–84)	77 (74–80)	78 (74–81)	82 (77–85)	82 (77–86)
	2-year	63 (60–66)	60 (56–64)	64 (59–67)	67 (62–71)	73 (68–78)
	3-year	49 (46–52)	49 (45–53)	52 (48–56)	60 (55–64)	67 (61–72)
	4-year	40 (37–43)	40 (36–44)	47 (42–51)	55 (49–60)	61 (55–67)
	5-year	33 (30–36)	36 (32–40)	42 (38–47)	50 (44–55)	57 (50–63)
	6-year	29 (26–33)	33 (29–36)	39 (34–43)	46 (41–52)	55 (48–61)
	7-year	27 (24–30)	30 (26–34)	36 (31–41)	44 (39–50)	50 (42–57)
	8-year	24 (21–28)	28 (24–32)	35 (30–39)	41 (34–47)	50 (42–57)

To illustrate how to interpret the table: If the time already survived is 5 years (column) the chance of 1- to 8-year survival from that time-point is presented in the row. E.g. if a patient has survived 5 years (column) the 1-year conditional survival from that time is 82% (row) and the 8-year conditional survival from that time point is 50% (row).

OS: overall survival; CI: confidence interval.

**Figure 2:**
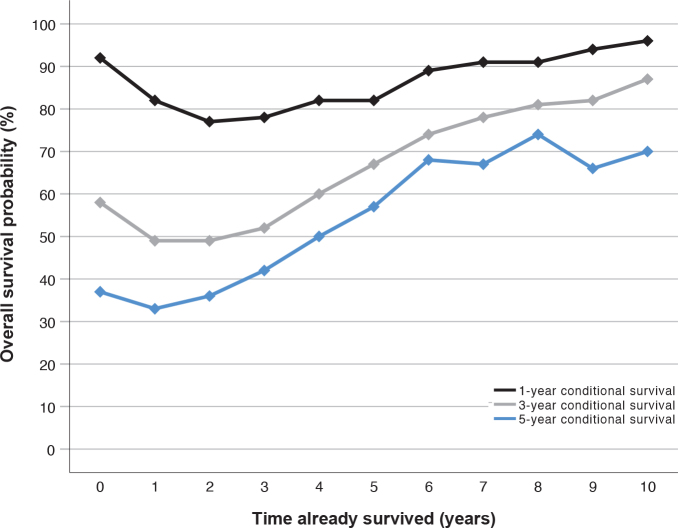
One-, three- and five-year conditional survival of patient diagnosed with advanced ovarian cancer between 2009 and 2018 in the Stockholm/Gotland Region, Sweden. Conditional overall survival estimates the probability of surviving (y-axis) one (black line), three (light grey line), and five (dark grey line) more years depending on how many years a patient has already survived (x-axis). For example, if the time survived is 4 years (x-axis), the conditional 1-year survival (black line) is ~80% (y-axis), the conditional 3-year survival (light grey line) is ~60%, and the conditional 5-year survival is ~50%.

The 1-, 3- and 5-year conditional survival by time already survived is graphically displayed in Figure 2.

A total of 76 (8.5 %) patients were alive >10 years after diagnosis (see Supplementary Table 2). The majority had FIGO stage III (87%, *n* = 66), ASA score I-II (68%, *n* = 52), upfront surgery (78%, *n* = 59), no residual disease (82%, *n* = 62), and a low surgical complexity score (40%, *n* = 53). Moreover, almost all were in complete remission after first line treatment (92%, *n* = 70) (see Supplementary Table 2).

**Figure 3:**
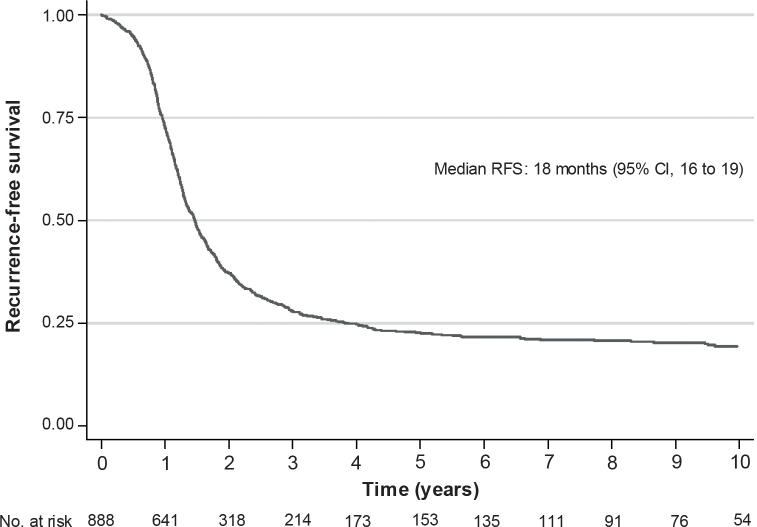
Recurrence-free survival in patients diagnosed with advanced ovarian cancer between 2009 and 2018 in the Stockholm/Gotland Region, Sweden. RFS: recurrence-free survival; CI: confidence interval.

In all, 740 (83%) patients experienced a recurrence; details on treatment at time of relapse are presented in Supplementary Table 3. The median recurrence-free survival was 18 months (95% CI: 16 to 19) (see Figure 3). The corresponding numbers by stage, by complete macroscopic resection or complete remission after first line treatment are presented in Supplementary Figure 1.

The overall survival after first recurrence is presented in Supplementary Figure 2 and by platinum free interval, stage, complete macroscopic resection or complete remission after first line treatment in Supplementary Figures 3.

The association between clinical factors and hazard of recurrence is presented in Table 3. In the adjusted analysis, interval debulking surgery (HR: 1.36; 95% CI: 1.10 to 1.70; *p* = 0.01), a high surgical complexity score (HR: 1.67; 95% CI: 1.34 to 2.09, *p* < 0.001), size of residual tumour (≤10 mm HR: 1.81; 95% CI: 1.45 to 2.25; *p* < 0.001 and >10 mm; HR: 2.15; 95% CI: 1.65 to 2.80; *p* < 0.001), and evidence of disease at end of first line treatment (HR: 2.40; 95% CI: 1.97 to 2.93; *p* < 0.001) increased the hazard of recurrence.

**Table 3:** Association between clinical factors and recurrence free survival in patient diagnosed with advanced ovarian cancer between 2009 and 2018 in the Stockholm/Gotland Region, Sweden.

Variable	No. (%)	Unadjusted^a^		Adjusted^a,b^	
		HR (95% CI)	*p*-value^c^	HR (95% CI)	*p*-value^c^
FIGO stage					
III	611 (69)	1	1	1	
IV	277 (31)	1.55 (1.32–1.81)	**< 0.001**	1.14 (0.96–1.35)	0.14
Timing of surgery					
Upfront	664 (75)	1		1	
Interval	224 (25)	1.57 (1.33–1.85)	**< 0.001**	1.36 (1.10–1.70)	**0.01**
Surgical complexity score^d^					
Low (0–3)	387 (44)	1		1	
Medium (4–7)	231 (26)	0.59 (0.48–0.71)	**< 0.001**	1.01 (0.80–1.28)	0.96
High (≥ 8)	268 (30)	0.97 (0.82–1.16)	0.77	1.67 (1.34–2.09)	**< 0.001**
Residual tumour, mm					
0	513 (58)	1		1	
≤ 10	163 (18)	2.42 (1.99–2.95)	**< 0.001**	1.81 (1.45–2.25)	**< 0.001**
> 10	208 (23)	3.22 (2.69–3.86)	**< 0.001**	2.15 (1.65–2.80)	**< 0.001**
Complete remission at end of first line treatment					
Yes	523 (59)	1		1	
No	361 (41)	3.85 (3.29–4.51)	**< 0.001**	2.40 (1.97–2.93)	**< 0.001**

HR: hazard ratio; CI: confidence interval; FIGO: International Federation of Gynecology and Obstetrics; ASA-score: American Society of Anaesthesiologists physical status classification.

a The variables age, ASA-score, and number of cycles of chemotherapy did not meet the proportional hazards assumption and were included as strata, why not reported separately in neither the univariable nor multivariable analyses.

b Adjusted for age, ASA-score, FIGO stage, timing of surgery, surgical complexity score, residual tumour, number of cycles of chemotherapy, and complete remission at end of first line treatment.

c Walds *p*-value.

d According to Aletti et al. [20].

## Discussion

Our study indicates that the 3- and 5-year conditional survival rates improve during the initial 5 years following a diagnosis of advanced ovarian cancer. In addition, approximately one-tenth of women achieve very long-term survival. Moreover, most patients experience recurrence within a year after completing first-line treatment.

Albeit prognosis after a cancer diagnosis may never be absolute, it is an essential variable to consider for both patients and caregivers to plan for surveillance and treatment. In diagnosis with a generally dismal prognosis, like in advanced ovarian cancer, counselling of patients is particularly challenging and information on prognosis may risk inflicting additional anxiety. Nevertheless, the expected survival may change with the passing of time, that is conditioned by the time already survived. Previous studies examining conditional survival in ovarian cancer are limited and include all stages [22–24]. As survival is generally very good in early-stage ovarian cancer, these studies may not be considered representative for the majority of women with advanced ovarian cancer [4]. Nevertheless, it is encouraging that our results, suggest that conditional survival improves over the first 5 years after diagnosis.

Prognostic factors associated with longer survival in ovarian cancer are well-established [5–8]. However, the factors influencing very long-term survival (>10 years) remain largely unknown. In our study, the median age of very long-term survivors was close to the median of the whole cohort, suggesting limited benefit of younger age. As anticipated, almost all very long-term survivors had undergone complete macroscopic resection, exhibited lower tumour burden, and were assessed as being in complete remission following first-line treatment, consistent with previous findings [8]. However, we were surprised to find that this group also included patients with residual tumour and those without complete remission. These results reveal the clinical heterogeneity of very long-term survival in ovarian cancer, complementing previous genomic studies in which no definitive genetic signature could be identified [7]. Aligning with previous reports, 8.6% of women in our study survived beyond 10 years [4, 25]. However, this estimate may be overstated, as our cohort excludes the significant proportion of patients with advanced ovarian cancer who do not undergo surgical treatment altogether [13, 15].

As expected, in line with previous studies, most patients experienced a recurrence [9–12]. The risk factors for recurrence were anticipated and largely consistent with those associated with prognosis (e.g. residual tumour, timing of surgery, tumour burden). Most patients with recurrence received second-line treatment including platinum-based chemotherapy. A longer platinum-free interval conferred the best survival (Supplementary Figure 3A) as previously suggested [1, 9, 26]. Of patients with recurrence, 11% (*n* = 83) were platinum refractory and progressed during first line treatment and a quarter (23%, *n* = 167) were platinum resistant, with recurrence within 6 months of completion of first line treatment (Supplementary Table 3), also consistent with previous reports [9, 27].

The effect of secondary cytoreductive surgery in addition to chemotherapy at the time of first recurrence has been assessed in three randomised trials, yielding conflicting results [28–30]. In the present study, 8% (*n* = 58) of patients with recurrence underwent secondary cytoreductive surgery. Whether this figure may be considered high or low remains uncertain, as the period-prevalence of secondary cytoreductive surgery following advanced ovarian cancer in a public healthcare system setting has not been reported previously.

Our study is limited by its observational design, leaving inherent possibilities for systematic error. In addition, by excluding patients receiving non-surgical treatment, the estimates do not capture all patients with advanced ovarian cancer. However, we report a unique material representing the whole accounted for and surgically treated population of patients with advanced ovarian cancer (not a sample of the population) in the Region of Stockholm/Gotland, Sweden. By utilising the Swedish population registries and unique personal identification numbers to cross-check the registries, there was no loss to follow-up. In addition, known potential confounders and covariates were controlled to the best of our ability. Nevertheless, the generalisability may only extend to similar settings.

In conclusion, our results suggest that the 3- and 5-year conditional survival improves significantly with time survived following an advanced ovarian cancer diagnosis. In addition, very long-term survival may be achieved. Furthermore, most patients experience relapse within 1 year after completing first-line treatment. These data may be used to improve patient counseling, inform surveillance strategies, and serve as a benchmark for future research.

## Author contributions

S. Salehi and N. Groes Kofoed contributed to the study design and idea. S. Salehi for data acquisition. N. Groes Kofoed and S. Salehi updated the dataset. M. Bottai and N. Groes Kofoed performed the statistical analysis. All authors analysed and interpreted the data. S. Salehi and N. Groes Kofoed prepared the first draft of the manuscript. All authors commented, edited, reviewed, and finally approved the last version of the manuscript.

## Supplementary Material

The trajectory of conditional, recurrence-free, and long-term survival in a complete 10-year cohort of patients with advanced ovarian cancer

## Data Availability

Data on an individual level cannot be shared according to ethical review board. However, data on an aggregated level can be shared upon reasonable request.
