# Supplementary material for: The trajectory of conditional, recurrence-free, and long-term survival in a complete 10-year cohort of patients with advanced ovarian cancer
Source: Acta Oncol. 2025 Mar 17;64:42994. doi: 10.2340/1651-226X.2025.42994 (PMC11931853; doi:10.2340/1651-226X.2025.42994)

Supplementary material has been published as submitted. It has not been copyedited, or typeset by Acta Oncologica

Supplementary Table 1. Follow-up and completeness of data

**Sample size (*n*)**	888
**Number of events**[a]	688
**Accrual period**	Jan 2009 - Dec 2018
**Closing date**	20 June 2024
**Median follow-up, years (min-max)**	
**Maximum**[b]	10.5 (5.1-15.5)
**Potential**[c]	3.6 (0.01-15.2)
**Observed**[d]	3.6 (0.01-15.2)
**Clark's completeness index**	100%

[a]Death. [b]Time from surgery to closing date irrespective of whether an event occurred. [c]Time from surgery to closing date or death. [d]Time from surgery to closing date, death or loss to follow-up.

Supplementary Table 2. Clinical and treatment characteristics of very long-term survivors of advanced ovarian cancer diagnosed between 2009-2018, in the Stockholm Gotland Region, Sweden.

**Characteristics**	Long-term n=76
Age, years	
Median (IQR*)	64 (54-69)
Age, years group, no. (%)	
≤59	29 (38)
60-69	29 (38)
≥70	18 (24)
FIGO stage, no. (%)	
III	66 (87)
IV	10 (13)
ASA-score, no. (%)	
I-II	52 (68)
III-IV	16 (21)
Missing	8 (11)
Timing of surgery, no. (%)	
Upfront	59 (78)
Interval	17 (22)
Surgical complexity score[a], no. (%)	
Low (0-3)	40 (53)
Intermediate (4-7)	25 (33)
High (≥8)	11 (15)
Residual tumour, no. (%)	
0 mm	62 (82)
≤ 10 mm	8 (11)
>10 mm	6 (8)
Number of cycles of chemotherapy	
Median (IQR)	6 (6-7)
Complete remission at end of first line treatment, no. (%)	
Yes	70 (92)

No	6 (8)

Abbreviations: IQR, Interquartile range; FIGO, International Federation of Gynecology and Obstetrics; ASA-score, American Society of Anaesthesiologist physical status classification.

[*]Refers to 1st to 3rd quartile.

[a]According to the Aletti et al (20).

Supplementary Table 3. Treatment characteristics after recurrence in women diagnosed with advanced ovarian cancer between 2009-2018 in the Stockholm/Gotland region, Sweden

**Characteristics**	n=888
Recurrence, no. (%)	
Yes	740 (83)
No	135 (15)
Death during primary treatment[a]	5 (1)
Missing	8 (1)
Platinum-free interval after completion of first-line chemotherapy, no. (% of patients with recurrence)	
Progression during primary treatment	76 (10)
<6 months	167 (23)
6-12 months	223 (30)
>12 months	267 (36)
Death due to progression during primary treatment	7 (1)
Secondary cytoreductive surgery at recurrence, no. (% of patients with recurrence)	
Yes	58 (8)
No	680 (92)
Missing	1 (0)
2nd line treatment, no. (% of patients with recurrence)	
Yes	636 (86)
No	103 (14)
Missing	1 (0)
Type of 2nd line treatment, no. (% of patients who received 2nd line treatment)	
Platinum-based	428 (67)
Doxorubicin-based	56 (9)
Paclitaxel	52 (8)
Cyclophosphamide (orally)	62 (10)
Other intravenous chemotherapy	21 (3)

Other oral anti-tumoral treatment	17 (3)
3rd line treatment, no. (% of patients who received 2nd line treatment)	
Yes	449 (71)
No	185 (29)
Missing	2 (0)
Type of 3rd line treatment, no. (% of patients who received 3rd line treatment)	
Platinum-based	104 (25)
Paclitaxel	91 (20)
Doxorubicin-based	50 (11)
Other intravenous chemotherapy	70 (16)
Cyclophosphamide (orally)	87 (19)
Other anti-tumoral treatment orally	36 (8)
Radiotherapy alone	1 (0)
PARP-inhibitor[b], no. (%)	
Yes	96 (11)
No	781 (88)
Missing	11 (1)
Bevacizumab, no. (%)	
Primary treatment	124 (14)
2nd line (% of patients who received 2nd line treatment)	34 (5)
3rd line (% of patients who received 3rd line treatment)	47 (10)

Abbreviations: PARP, poly-ADP-ribose polymerase.

[a]Three patients died from postoperative complications, one from anaphylactic reaction to chemotherapy and one had unknown cause without signs of progression on computer tomography prior to death.

[b]Any timing of treatment.

Supplementary Figure 1. Recurrence-free survival by stage, complete macroscopic resection and complete remission after first line treatment in patient diagnosed with advanced ovarian cancer between 2009-2018 in the Stockholm/Gotland Region, Sweden.
A: Whole cohort by stage. B. Patients with complete macroscopic resection by stage. C. Patients in complete remission at end of first line treatment by stage.
Abbreviations: RFS, Recurrence-free survival; CI, Confidence interval.

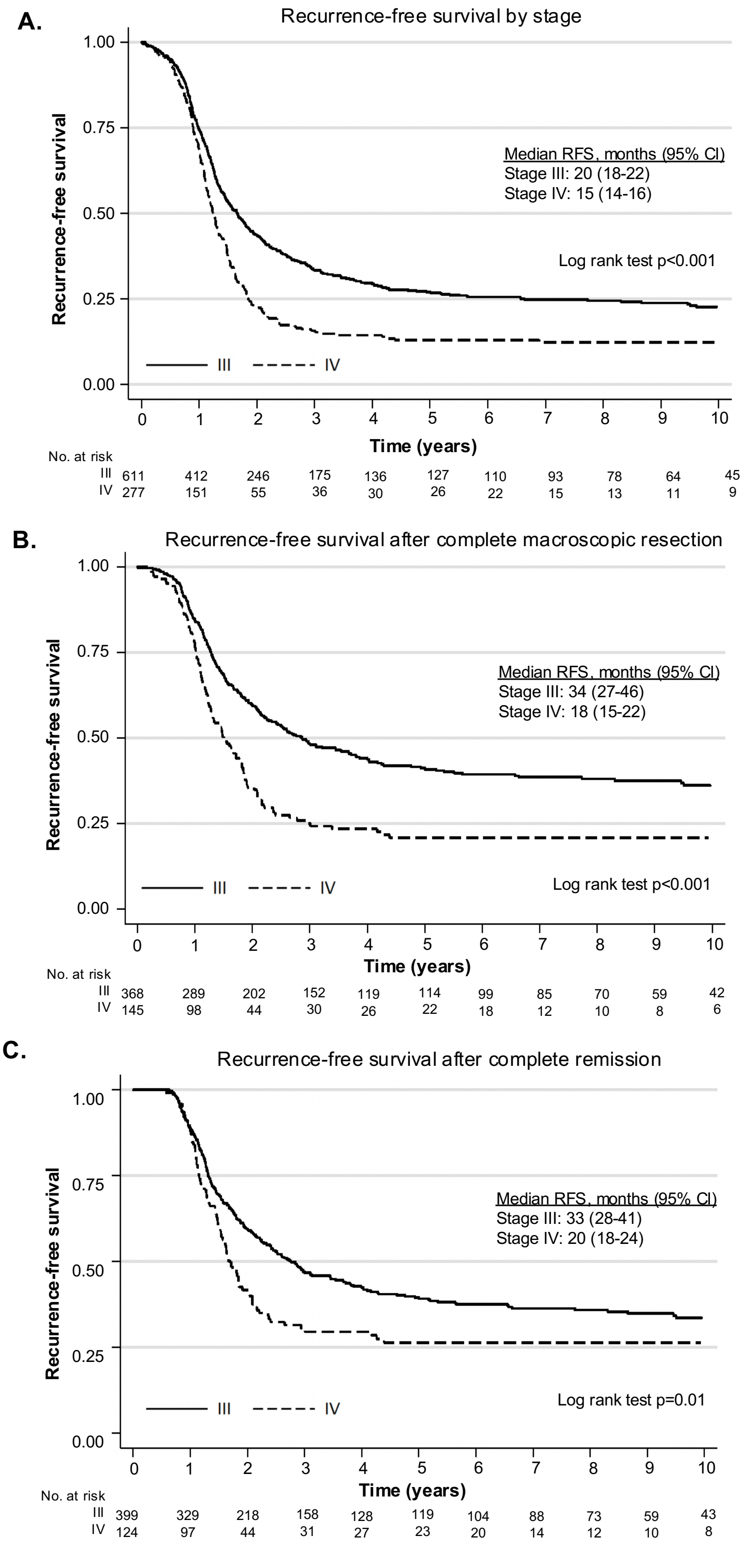

Supplementary Figure 2. Survival after **first recurrence in patients diagnosed with advanced ovarian cancer between 2009-2018 in the Stockholm/Gotland Region, Sweden.**

Abbreviations: OS, Overall survival; CI, Confidence interval.

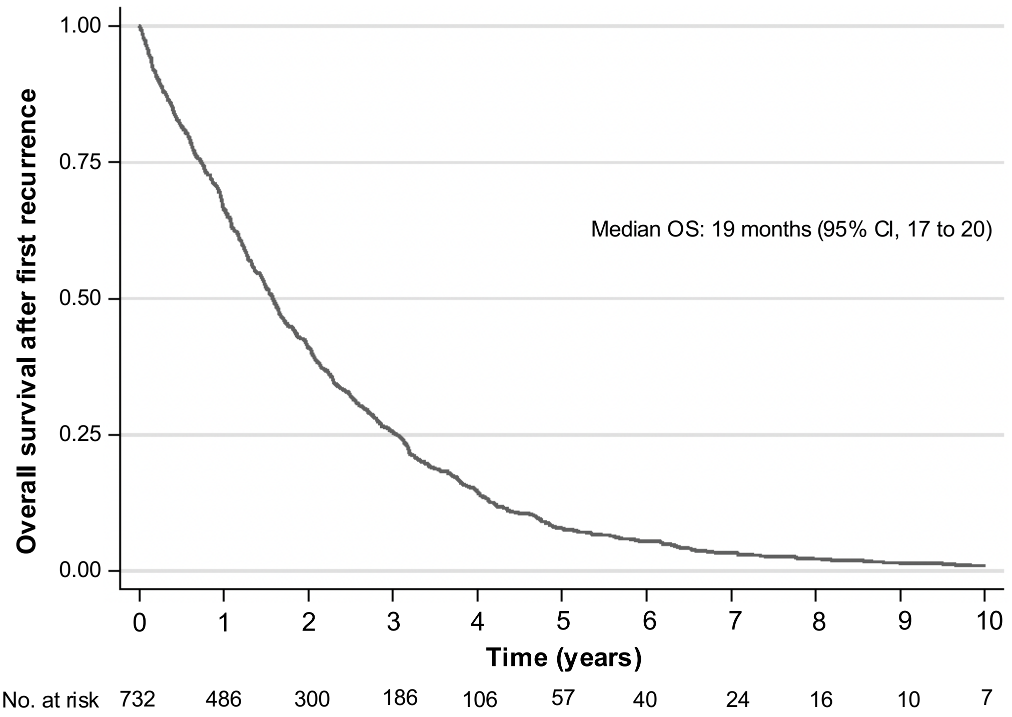

Supplementary Figure 3. Survival after **first recurrence by platinum-free interval, by stage, after complete macroscopic resection and complete remission after first line treatment in patient diagnosed with advanced ovarian cancer between 2009-2018 in the Stockholm/Gotland Region, Sweden.**

A: Platinum-free interval. B. Whole cohort by stage. C. Patients with complete macroscopic resection by stage. D. Patients in complete remission at end of first line treatment by stage.

Abbreviations: OS, Overall survival; CI, Confidence interval.

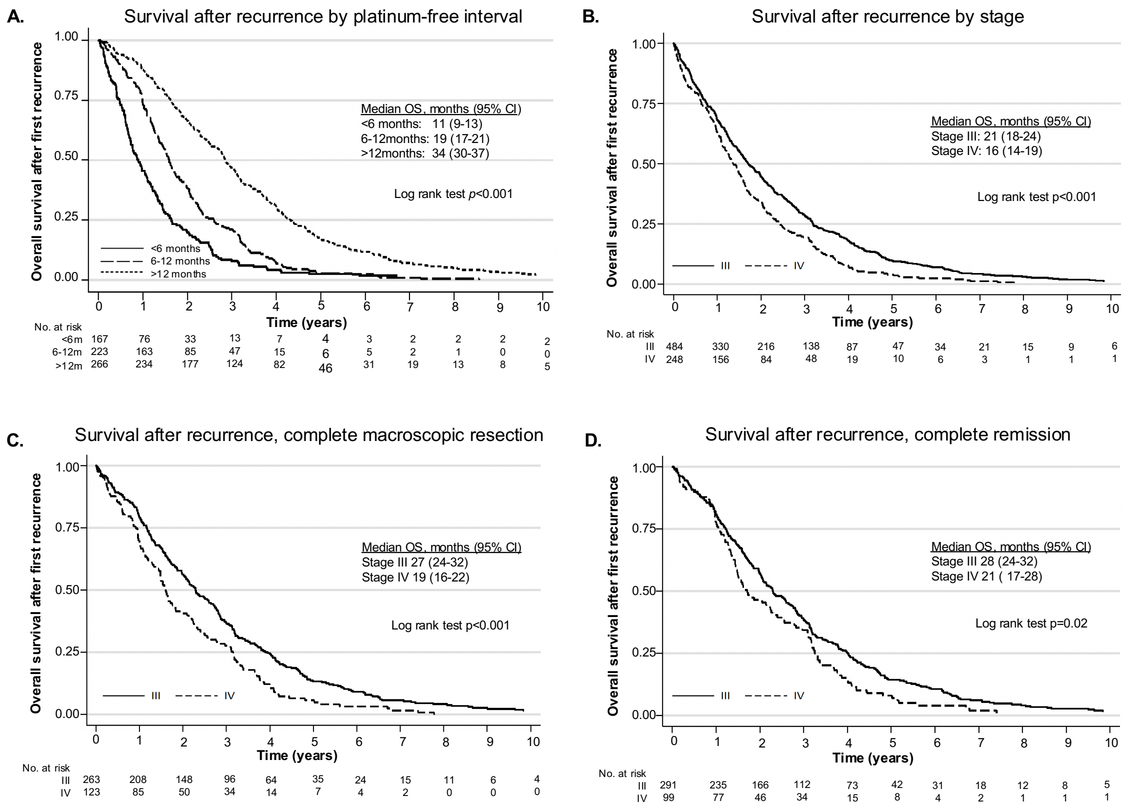

Supplement: The trajectory of conditional, recurrence-free, and long-term survival in a complete 10-year cohort of patients with advanced ovarian cancer [file AO-64-42994-s1.pdf]
